# Lesions of the Cervical Sympathetic Nerves

**Published:** 1905-02-18

**Authors:** 


### Hospital Clinics.

### LESIONS OF THE CERVICAL SYMPATHETIC NERVES.

Dr. Purves Stewart [1] is probably right in saying that for most practitioners the sympathetic nervous system represents little more than an echo of their student days. It is a mysterious department and its clinical shortcomings are little understood, but in its cervical segment, at all events, lesions of it supply a sufficiently definite clinical disturbance.

It may be well to be reminded of some anatomical facts, viz., that the system consists of two gangliated cords, situated in front of the vertebræ, and running from the base of the skull to the coccyx, all its fibres being involuntary. The rami communicantes, grey and white, connect it throughout its course with the anterior primary divisions of the spinal nerves. The grey rami are non-medullated; they start within the sympathetic cord, and, joining the anterior nerve-roots, send efferent fibres to the skin—vaso-motor, pilo-motor, and secretory—and also afferent branches by way of the posterior nerve-roots to the spinal cord. The white rami are medullated, and pass from the spinal nerves to the sympathetic ganglia. All the white rami consist of efferent fibres.

The cervical sympathetic contains important fibres: the oculo-pupillary, which supply the dilator pupillæ muscle, part of the levator palpebræ superioris, the orbital muscle of Müller, which keeps the eye forward in the orbit, the submaxillary gland, and the vaso motor apparatus and sweat-glands of the head and neck. The course of these fibres is peculiar, and clinically important. Rising from the pupil-dilating centre in the medulla oblongata, they pass down in the lateral columns of the spinal cord to the "cilio-spinal centre" in its lower cervical region. Hence, they leave the spinal cord by the white rami communicantes from the first and second dorsal nerves, and, joining the inferior cervical ganglion of the sympathetic, run upward to the orbit behind the jugular vein.

These pupillary fibres are thus liable to be involved in any part of their long course, a point of high clinical significance, since even strictly spinal-cord lesions such as those of syringo-myelia can produce the symptoms of cervical sympathetic paralysis.

These symptoms are—(1) No dilatation of the pupil in a dim light; (2) partial drooping of the upper lid; (3) enophthalmos from paralysis of the muscle of Müller; (3) absence of the cilio-spinal reflex—that is, absence of the pupil-dilation which usually follows upon pinching the skin of the neck on the same side; (4) a diminished intra-ocular tension; (5) absence of sweating on the area of skin supplied by the cervical sympathetic.

The classes of lesion liable to produce this condition of cervical sympathetic paralysis are well exemplified by the cases adduced for illustration. Thus, in one case a woman underwent an operation for the removal of deep glands of the neck. After the operation she was informed that the jugular vein had been wounded. She herself noted that she was unable completely to open the eye on the affected side, and that when she flushed the flush was limited to the opposite side of her face. It was clear that the cervical sympathetic had been wounded with the jugular vein.

Again, a soldier was shot in the neck during the South African war. On recovery it was found that he also had the typical ocular symptoms, and, the climate being tropical, Dr. Stewart was able, by an ingenious and simple device, accurately to define the area of skin supplied with sweat-secreting fibres by the cervical sympathetic. By blowing powdered charcoal on to the skin the sweating surface was clearly defined by adhesion of the particles. It was found to be limited by the middle line of the face in front and the middle line of the neck behind. Downwards it reached the middle line as before, both in front and behind, and circled the chest at the level of the third rib in front and the spine of the scapula behind. The whole of the upper extremity on the affected side was included in the sweatless area.

A third case was that of a lad who presented the signs of this paralysis on one side, and who, on examination, was found to have a tumour growing at the apex of the chest, which accounted for the lesion of the sympathetic. In this connection the author observes that even old apical tuberculous scars may produce the symptoms of sympathetic paralysis to some degree.

A fourth case was that of an epileptic who in the course of a seizure fell and ruptured the whole brachial plexus on one side. In this case the oculo-pupillary fibres were involved in the lesion of the first and second dorsal nerve roots.

It is well to remember that the pressure of a tumour may give rise in the first instance to stimulation of the sympathetic, in which case the symptoms will of course be reversed—that is to say, there will be exophthalmos and dilatation of the pupil, etc., but in such cases a paralytic stage usually supervenes in the course of time.

[1] Pract., Feb. 1905.

